# Environmental and perceived stress in Australian dental undergraduates: Preliminary outcomes

**DOI:** 10.15171/joddd.2016.043

**Published:** 2016-12-21

**Authors:** Shannon Astill, Nikelle Ricketts, Love-Amrit Singh, Dylan Kurtz, Yong Hoon Gim, Boyen Huang

**Affiliations:** ^1^Westfund Health, Mackay, Australia; ^2^School of Medicine and Dentistry, James Cook University, Cairns, Australia; ^3^Eden Dental Surgery, Eden, Australia; ^4^School of Dentistry and Health Sciences, Charles Sturt University, Orange, Australia

**Keywords:** Dental environmental stress, perceived stress, dental students, dental education

## Abstract

***Background.*** Dental students have reported a high prevalence of psychological stress and the causes are associated with the challenging dental environmental and demographic factors. This study aimed to conduct a preliminary investigation on dental students’ stress status, using a sample of first-to-third-year Bachelor of Dental Surgery students in an Australian university. Special interests included causes of dental environmental stress and access to help services.

***Methods.*** A sample of 145 students was surveyed with a modified Dental Environmental Survey and Depression Anxiety Stress Scale in 2014. The participants’ demographic information was also collected.

***Results.*** The response rate was 95.4%. Second-year (P = 0.042), third-year (P < 0.001) and employed students (P = 0.027) were more likely to report stress resulting from transition to clinical learning. Third-year students were more often stressed about communicating and approaching staff (P = 0.023) as well as different opinions between staff (P < 0.001) and reduced holidays (P < 0.001). Students that were younger than 21 years of age (P = 0.001), that were first years (P < 0.001), and that were not in a relationship (P = 0.010) more often found difficulty of course work stressful. Students who were not in a relationship more often considered learning manual dexterity a source of stress (P = 0.034). Students previously seeking professional help were more likely to be stressed (P = 0.010).

***Conclusion.*** Causes of dental environment stress varied among years of study and demographic backgrounds. Professional support to stressed students should be enhanced. Further investigation is indicated.

## Introduction

Psychological stress is defined as strain or pressure that can be adaptive or debilitating.^1^ Excessive stress can lead to various medical complications and increase the risk of mental illness.^2-4^ Students in tertiary education have been found to exhibit high levels of perceived psychological stress due to difficulty in transition to higher education, with the mean age of university students being a risk factor for mental health.^2,5^ Dentists, and dental students in particular, have reported a high prevalence of stress.^6^

The causes of psychological stress in dental students are multi-factorial and numerous; however, they are associated with the challenging dental environmental and demographic factors.^6,7^ The way in which perceived stress levels were assessed independently varies in the literature. Multiple studies have used the Perceived Stress Scale (PSS)^6,8^ and the Depression Anxiety and Stress Scale (DASS)^9-14^ to assess perceived stress levels. Both the DASS and PSS have been shown to demonstrate sound psychometric properties, good internal consistency and reliability.^9,15^However, previous Australian research on dental students’ stress has used the PSS and not the Australian developed DASS.^9,16^ This represents a gap in literature with the use of the DASS on dental students.

The majority of studies that assessed dental environmental stress on dental students used the Dental Environmental Stress (DES) survey.^3,6,17^ Although having been used more recently internationally, the DES survey has not been used within Australia for the past 12 years, with the last study surveying students in Adelaide.^6^ This highlights a gap in the literature for stress levels of dental students in Australia, especially in dental students outside of a capital city or those in a newly established Bachelor of Dentistry (BDS) course. Studies have reported that academic and clinical performance was among the highest stressors of DES.^6,17-20^ However, perceived stressors varied considerably among students from different dental schools and across different year levels.^6,21,22^ The reason for this variation can be attributed to the accumulative nature of academic and clinical work through the course.^3,6^ Students in their first year of dental school were more likely to be stressed about academics, whereas students in higher levels were more stressed about clinical work.^21^ Studies have indicated that the reason why stress rose was the increase in the amount of clinical coursework.^6^ Although implementation of DES has been reported, inconsistencies were identified. This highlights a gap in the knowledge about DES in Australian dental schools.

Demographic factors such as age, gender, marital status and work can be associated with DES levels, as well as perceived stress. As young adults are more likely to be reported as having a higher rate of mental illness,^5^ and as the majority of students in a BDS are young adults, it is more likely for BDS students to have a mental illness due to psychological stress. Female dental students tended to perceive themselves as being more stressed than males, with differences between the influences of environmental stress between genders.^23^ However, this conclusion was not consistent through the literature.^6,8,21,23-25^ Previous studies reported an association between marital status and perceived stress levels, identifying a higher frequency of stress in those who are married.^6,24^ Literature has reported a relationship between funding status and perceived stress levels. The limited literature identified also indicated a gap in knowledge with regards to students who work while studying.^8,23-27^

The onset of mental illness, such as clinical depression and various anxiety disorders, has a strong link with excessive amounts of stress.^2-4^ The success of help services to manage mental illness and psychological stress is well established. Nevertheless, current research on mental health service use by dental students is very limited globally.^28,29^ This represents a significant need to establish if help services are accessed by dental students.

In order to enhance the understanding of dental students’ stress, the aim of this study was to conduct a preliminary investigation on first-to-third-year BDS students’ stress status in an Australian university. Focuses of the study included causes of DES, students’ self-perceived stress levels and to identify whether students had accessed help services.

## Methods

Prior to commencement of this study, appropriate ethics approval was obtained from the James Cook University (JCU) Human Research Ethics Committee (Reference ID: H5617). Participants for this study were required to be enrolled in the BDS and studying full time. Due to the researchers’ being part of the fourth-year course at the research time, this group was excluded from the study to ensure anonymity of participants. Fifth years were also excluded, as they were allocated to various locations across Australia and their participation was difficult at the time of study.

The assessment of dental student stress was predominantly achieved through the combination of the DES survey and the DASS survey tools, both of which have been used in various studies.^8,10,11,15,17,22,24,26,30-32^ The DES and DASS were shortened due to the preliminary nature of the study, with questions being included in accordance with their relevance to the cohort studying the BDS at JCU.

The original short version of the DASS consisted of 21 items, including three self-reported scales which are designed to measure depression, anxiety and stress.^33^ This survey has been modified and used in a number of studies.^10,11,15,32^ In this study, the short version of the DASS was modified to focus on the stress components of the survey, as seen in Table 1. The modified DASS used in the study included 7 stress-related questions, recorded on a 4-point Likert scale, with participants categorizing their stress as 0 (Did not apply to me at all), 1 (Applied to me to some degree, or some of the time), 2 (Applied to me to a considerable degree, or a good part of time) and 3 (Applied to me very much, or most of the time). The scores were then added up and multiplied by 2 to be able to be correlated with the original surveys interpretation table as it had 14 questions on stress.^33^

**Table 1:** Items included in the modified DASS and DES surveys

**DASS items**	
	
1	I found it hard to wind down
2	I tended to over-react to situations
3	I felt that I was using a lot of nervous energy
4	I found myself getting agitated
5	I found it difficult to relax
6	I was intolerant of anything that kept me from getting on with what I was doing
7	I felt that I was rather touchy
	
**DES items**	
	
8	Moving away from home
9	Having reduced holidays
10	Communicability and approachability of staff
11	Amount of assigned coursework
12	Difficulty of coursework
13	Concerns about learning precision of manual dexterity
14	Transition from preclinical to clinical work
15	Differences in opinion between staff concerning procedures within the teaching and clinical setting

On the other hand, the DES survey is a closed-ended questionnaire, consisting of 38 questions categorized into the areas of living accommodation, interpersonal relationships, academics, clinical skills and miscellaneous.^22^ This survey has been modified and used in a number of studies.^8,10,11,15,17-22,24,25^ The DES questionnaire used in this study was reduced to 8 items, including the categories of living accommodation, academic, clinical skills and miscellaneous, as seen in Table 1. The modification reflected the relevance to the JCU BDS cohort and highly stressful areas by previous dental students.^34^ The participants assessed the stressors on a 4-point Likert scale categorized as 0 (Not stressful), 1 (Slightly stressful), 2 (Moderately stressful) and 3 (Severely stressful). In addition, a single polar question of whether students accessed help services was included in the survey. The inclusion of this question allowed the study to identify whether stressed students had accessed help services to cope. Of further note, demographic data collected included gender, age, year of degree, relationship status and employment status.^34^

Data entry and statistical analyses were carried out using IBM SPSS Statistics for Windows (Version 22.0. Armonk, NY: IBM Corp). Frequency distributions of gender, age, year groups, relationship status, employment status and history of accessing counseling services were conducted. A binary logistic regression method was carried out to test the association of DES scores and DASS factors with demographic factors. The main outcome is whether a student was stressed or not, according to the DES scores defined by the developers. The predictors were selected with a backward mode. Due to the small sample size and the nature of a preliminary study, Hosmer-Lemeshow test was not used in this study. One-way analysis of variance (ANOVA) was also applied to examine the difference of individual DES and DASS items according to age, gender, year of study, relationship status and employment status. The level of significance was set at 5%.

## Results

**Figure 1.**
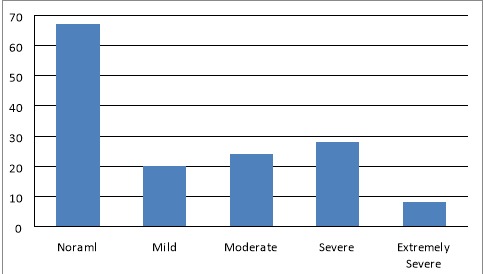
Frequency distribution of outcomes of individual stress status according to individual DASS score (n = 145).

A total of 252 JCU BDS students were invited to participate based on the registration records of the dental school in the academic year 2014. The questionnaires were handed out to students attending designated lecture times. Among the 152 students present at the lectures, 145 completed a questionnaire and this contributed to a response rate of 95.4%. The final sample included 50 (34.5%) year one, 41 (28.3%) year two and 54 (37.2%) year three students. Male students made up 70 of the participants (48.3%), and 75 (51.7%) were female. Ninety-nine (68.3%) students were under the age of 21, whilst the other 46 (31.7%) were 21 years of age and older. Seventy-eight (53.8%) students perceived themselves as stressed although 67 (46.2%) did not (Figure 1). The mean of the DASS scores was 16.9±9.9. It was found that those who were receiving professional help were more likely to be stressed (F = 4.73, P = 0.010, Table 2). Age, gender, relationship status, employment status and year level were not associated with the DASS score (P ≥ 0.087) (Table 2). Students were separated into stressed and not stressed based on their DASS score, as can be seen in Table 3. Dental students who had a higher DASS score more often sought professional help (P = 0.029, OR = 4.27, 95% CI: 1.16–15.69) (Table 3). As indicated in Table 3, age, gender, relationship status, employment status and year of dental education were not associated with students’ DASS scores (P ≥ 0.073).

**Table 2:** One-way ANOVA analysis of individual DASS items

	**No.**	**Mean DASS Score**	**F**	**P-value**
**Age**			2.97	0.087
Under 21	99	17.88		
21 and Older	46	14.89		
**Gender**			2.05	0.155
Male	70	15.74		
Female	75	18.08		
**Professional help**			4.73	0.010^*^
Yes	17	23.65		
No	128	16.02		
**Relationship status**			1.34	0.265
In a relationship	62	18.13		
Not in a relationship	83	15.90		
**Employment status**			1.447	0.231
Not employed	85	16.07		
Employed	60	18.07		
**Dental year**			0.752	0.473
First Year	50	16.16		
Second Year	41	16.15		
Third Year	54	18.23		

^*^P<0.05

**Table 3:** Frequency distribution of demographics, dental year levels, receiving professional help, employment status, relationship status compared to stress status (n = 145)

	**Stressed**	**Not Stressed**	**All**	**OR (95% CI)**	**P-value**
**Age**					
Under 21	58 (58.6%)	41 (41.4%)	99 (68.3%)	1	
21 and Older	20 (43.5%)	26 (56.5%)	46 (31.7%)	1.84(0.91 – 3.73)	0.091
**Gender**					
Male	32 (45.7%)	38 (54.3%)	70 (48.3%)	1	
Female	46 (61.3%)	29 (38.7%)	75 (51.7%)	1.83(.95 – 3.56)	0.073
**Dental Year**					
First year	26 (52.0%)	24 (48.0%)	50 (34.5%)	1	
Second year	22 (53.7%)	19 (46.3%)	41 (28.3%)	1.07(0.47 – 2.45)	0.875
Third year	30 (55.6%)	24 (44.4%)	54 (37.2%)	1.15(0.53 – 2.50)	0.716
**Receiving professional help**				
Yes	14 (82.4%)	3 (17.6%)	17 (11.7%)	1	
No	64 (50.0%)	64 (50.0%)	128 (88.3)	4.27 (1.16 – 15.69)	0.029^*^
**Employment status**					
Employed	32 (53.3%)	28 (46.7%)	60 (41.4%)	1	
Not employed	46 (54.1%)	39 (45.9%)	85 (58.6%)	0.85 (0.55 – 2.07)	0.850
**Relationship status**					
Not in a relationship	42 (50.6%)	41 (49.4%)	83 (57.2%)	1	
In a relationship	36 (58.1%)	26 (41.9%)	62 (42.8%)	0.74 (0.38 – 1.44)	0.370

^*^P<0.05

**Figure 2.**
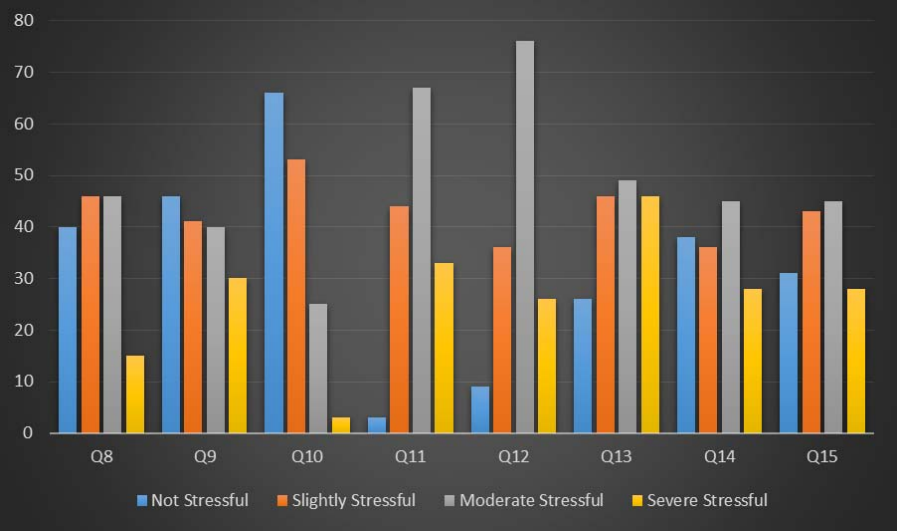
Frequency distribution of outcomes of individual stress status according to DES questions (n = 145).

On the other hand, female students had an increased likelihood of finding moving away from home stressful (F = 9.77, P = 0.002, Table 4). Second- and third-year students demonstrated a higher likelihood of stress regarding the reduction in holidays compared with first-year students (F = 18.48, P < 0.001, Table 4). Students that were 21 years of age or older (F = 12.42, P = 0.001), that were in their first year of study (F = 9.22, P < 0.001), and that were not in a relationship (F = 4.79, P = 0.010) were more likely to find the difficulty of course work stressful (Table 4). Students not in a relationship were more likely to be stressed concerning manual dexterity (F = 3.54, P = 0.032, Table 4). Third-year students had an increased probability of stress concerning the transition from preclinical to clinical work when compared to those in the first and second years (F = 8.99, P < 0.001, Table 4). Third year students were more likely to be stressed regarding the differences in opinions between staff concerning procedures than first- and second-year students (F = 18.30, P < 0.001, Table 4). Figure 2 illustrates the mean DASS scores of individual DASS items.

**Table 4:** One-way ANOVA analysis of individual DES items

	**No.**	**Mean DES Score**	**F**	**P-value**
**Relocation**			9.77	0.002^*^
Male	70	0.99		
Female	75	1.47		
**Reduced holidays**			18.48	<0.001^*^
First Year	50	0.90		
Second Year	41	0.95		
Third Year	54	1.04		
**Difficulty of coursework**			12.42	0.001^*^
Under 21	99	2.00		
21 and Older	46	1.55		
			9.22	<0.001^*^
**First year**	50	2.18		
Second Year	41	1.61		
Third Year	54	1.62		
			4.79	0.010^*^
In a relationship	62	1.60		
Not in a relationship	83	1.95		
**Manual dexterity**			3.54	0.032^*^
In a relationship	62	1.30		
Not in a relationship	83	1.65		
**Clinical transition**			8.99	<0.001^*^
First Year	50	0.98		
Second Year	41	1.46		
Third Year	54	1.82		
**Staff inconsistency**			18.30	<0.001^*^
First Year	50	0.88		
Second Year	41	1.56		
Third Year	54	1.96		

^*^P<0.05

Furthermore, second-year (P = 0.019, OR = 3.13, 95% CI: 1.21–8.10) and third-year students (P < 0.001, OR = 6.20, 95% CI: 2.25–17.06) had higher probability to be stressed in regard to having reduced holidays. Third-year students also had higher probability to be stressed in regard to approachability and communicating with staff (P = 0.023, OR = 2.52, 95% CI: 1.14–5.56) (Table 5). First-year students were somewhat more likely to be stressed concerning manual dexterity (P = 0.051, OR = 0.36, 95% CI: 0.13–1.00, Table 5). In regard to transition from preclinical to clinical work, second-year (P = 0.042, OR = 2.55, 95% CI: 1.03–6.27) and third-year (P < 0.001, OR = 8.21, 95% CI: 2.81–34.00) students were more likely to be stressed (Table 5). The difference in opinion between staff about clinical procedures led to a higher probability of being stressed amongst second-year (P = 0.054, OR = 2.49, 95% CI: 0.99–6.28) and third-year (P < 0.001, OR = 37.80, 95% CI: 4.94–295.14) students (Table 5). As indicated in Table 6, students who were not in a relationship were more likely to be stressed concerning manual dexterity (P = 0.016, OR = 2.98, 95% CI: 1.23–7.22). Furthermore, those who were employed had a higher likelihood to be stressed due to transition from preclinical to clinical work (P = 0.027, OR = 2.50, 95% CI: 1.11‒5.66) (Table 6). Factors such as moving away from home, the amount of assigned coursework and difficulty of coursework were not related to significant DES scores (P ≥ 0.071).

**Table 5:** Frequency distribution of reduced holidays, approachability of staff, manual dexterity, transition from preclinical to clinical work, differences in opinion of staff and dental year level compared to stress level (n = 145)

	**Stressed**	**Not Stressed**	**All**	**OR (95% CI)**	**P-value**
**Reduced holidays**					
First year	28 (56.0%)	22 (44.0%)	50 (34.5%)	1	
Second year	33 (80.5%)	8 (19.5%)	41 (28.3%)	3.13 (1.21–8.10)	0.019^*^
Third year	48 (88.9%)	6 (11.1%)	54 (37.2%)	6.20 (2.25–17.06)	<0.001^*^
**Staff approachability**					
First year	23 (46.0%)	27 (54.0%)	50 (34.5%)	1	
Second year	19 (46.3%)	22 (53.7%)	41 (28.3%)	0.97 (0.43–2.22)	0.945
Third year	37 (68.5%)	17 (31.5%)	54 (37.2%)	2.52 (1.14–5.56)	0.023^*^
**Manual dexterity**					
First year	44 (88.0%)	6 (12.0%)	50 (34.5%)	1	
Second year	36 (87.8%)	5 (12.2%)	41 (28.3%)	0.96 (0.27–3.40)	0.950
Third year	39 (72.2%)	15 (27.8%)	54 (37.2%)	0.36 (0.13–1.00)	0.051
**Clinical transition**					
First year	27 (54.0%)	23 (46.0%)	50 (34.5%)	1	
Second year	31 (75.6%)	10 (24.4%)	41 (28.3%)	2.55 (1.03–6.27)	0.042^*^
Third year	49 (90.7%)	5 (9.3%)	54 (37.2%)	8.21 (2.81–34.00)	<0.001^*^
**Staff inconsistency**					
First year	27 (54.0%)	23 (46.0%)	50 (34.5%)	1	
Second year	31 (75.6%)	10 (24.4%)	41 (28.3%)	2.55 (1.03–6.27)	0.042^*^
Third Year	49 (90.7%)	5 (9.3%)	54 (37.2%)	8.21 (2.81–34.00)	<0.001^*^

^*^P<0.05

**Table 6:** Frequency distribution of stress status by concerns of manual dexterity in comparison to employment status and relationship status (n = 145)

**Clinical transition**					
	**Stressed**	**Not Stressed**	**All**	**OR (95% CI)**	**P-value**
**Employment status**					
Employed	50 (83.3%)	10 (16.7%)	60 (41.4%)	1	
Not employed	57 (67.1%)	28 (32.9%)	85 (58.6%)	2.50 (1.11–5.66)	0.027^*^
**Concerns of manual dexterity**					
Relationship status					
Not in a relationship	74 (89.2%)	9 (10.8%)	83 (57.2%)	1	
In a relationship	46 (74.2%)	17 (27.4%)	62 (42.8%)	2.98 (1.23–7.22)	0.016^*^

^*^P<0.05

## Discussion

This study demonstrated a higher likelihood of stress due to the transition from pre-clinical to clinical learning amongst second- to third-year dental students. A number of articles reported an increase in perceived stress as students progressed in the course, with spikes noted during the transition year.^20,24,25^ Nevertheless, a previous study reported that students without patient contact had a higher DES score.^19^ First-year students might find the transition from preclinical to clinical work less stressful as BDS students entered the clinical setting from the beginning of the third year. On the other hand, students who were employed tended to report stress related to the transition from pre-clinical to clinical work. This disagrees with some papers that have reported no correlation between working status and perceived stress.^24,25^ Since the transition from pre-clinical to clinical work can be in itself a stressor, the finding of this study is reasonable as the additional stress component of work could increase perceived stress levels.

Third-year students were more likely to be stressed about differences in opinion between staff concerning procedures. Although limited evidence of this finding was present in the literature, a study reported differences in opinions about the clinical staff being highly ranked stressors in third, fourth and fifth clinical years.^20^ This finding may be apparent due to the increased contact time with staff members in the clinical setting and differences becoming additive overtime. It is also useful in highlighting possible sources of frustration for students, as different supervisors often have different techniques that students are expected to adopt quickly to in order to treat patients.

Third-year students were more likely to report stress when communicating and approaching staff. Previous studies did not find a difference in the item between the years of dental study.^17,20^ This finding may result from the commencement to learn clinical professionalism in the third year of the BDS course, which is a challenging transition for students. As third-year students are in the practical part of their course, reporting stress may also suggest that the clinical portion requires more interactions or is more stressful overall. This could help show a particular area of stress that third-year students sustained.

Students under 21 years of age were more likely to report the difficulty of course work as a stress, as did first-year students. Due to the pressure of starting university and having to perform at a tertiary standard, first-year students that are often younger students would be more likely to find the difficulty of course work stressful. This disagreed with some papers that have reported no relationship between the difficulty of course work and age or year of course.^8,17,22^ This outcome highlights a risk group for stress which academic staff should be aware of through the curriculum to ensure that help services are available for younger and first-year students if they are struggling. This could also indicate a need to teach new students time management and coping strategies in order to overcome the difficulty of course work.

Moreover, first-year students more often considered learning manual dexterity as a source of stress. Dental students in their first year have only started to develop their clinical skills in the simulated learning environment. Students in their third year, on the other hand, are in the clinical part of the course, and consequently have more manual dexterity practice. Although it has been found that most dental students do perceive learning precision and manual skills as stressful, significant differences between years has not been identified.^17,20,22^ This finding may indicate the need for extra support and exercise provided to first-year students when they learn manual dexterity. By the time they reach the third year, this stress would have subsided due to the increase in practice.

Apart from first-year students, students not in a relationship were more often found to consider learning manual dexterity as stressful. Learning manual dexterity was not identified as stressful in other studies and no relationship was found between this and students’ relationship status.^17,20^ This study also found that these students were also more likely to be stressed about the difficulty of course work. This agreed with a previous study suggesting that single students were more stressed due to lack of time for relaxation than married students.^21^ This association should be further investigated to possibly identify an at-risk group.

This study reported that dental students who sought professional help were more likely to be stressed. A potential reason for the association between higher stress likelihood and seeking professional assistance is the causal relationship between the two variables. Nevertheless, data about the amount of help sessions, what type of help service the students accessed and the time sequence of stress and professional assistance were not attainable in this study. Thus, the higher stress likelihood might also have resulted from previous unsuccessful support the students sought. This could be justified with some past studies suggesting that stressed students did not receive the support required.^6,28,29^ Thus, this finding identifies a possible future area for research and indicates the need for better help services at dental schools.

This research demonstrated that second- and third-year students were more likely to be stressed about reduced holidays. This finding was consistent with previous studies which found reduced holidays as stressful,^6,20,22,31^ with only one study finding no significant difference between the year levels.^17^ The BDS course calendar showed that, as the course progresses, the overall amount of holidays decreases. This could suggest a direct correlation between reduced holidays and students in higher years. In addition, senior students have differing stressors as they move to more difficult coursework; however, this finding may also imply that students would prefer more holidays.^20^ The inefficiency of student study time in dental courses has also been found to increase perceived stress levels regarding reduced holidays.^6^ Further research could include fourth- and fifth-year students to evaluate if being more stressed about reduced holidays just occurs at the transition year, or if it is consistent in all the higher years. It is also necessary to take the potential covariance between variables such as reduced holidays and difficult course work into account for future investigations.

On the other hand, female students were more likely to be stressed about moving away from home. The literature has suggested relocation as a high perceived stressor.^6,20,22^ Past studies have shown that students who live at home had an overall reduced amount of perceived stress.^6,19^ The literature on the association between moving away from home and gender is limited. Therefore, this result may indicate a risk group which has not been properly identified. Appropriate support from dental schools to the additional risk group would be required.

This research has highlighted risk groups that are more likely to perceive themselves as stressed in regard to different areas of dental study. This information is useful not only as preliminary research, but also providing dental educators a better understanding when creating curriculum and interacting with students. This study also serves as a baseline for the current first-to-third years as they move through the BDS course. This research could be repeated as the cohorts’ move through the course, with changes in perceived stress, and its correlation with the DES, potentially being able to be observed as the course evolves.

The limitations of this preliminary study included modifying questionnaires to include less as well as a small sample size due to low attendance rates of classes when recruiting participants and not including higher year levels. Further investigation of the validity of the modified survey is indicated to enhance the reliability of the study method and to encourage participation.

## Conclusion

This research has served as an important baseline study to help fill the gaps in knowledge about perceived stress, DES and use of help services, along with correlations with demographic information of BDS students in Australia. This research found that people who sought professional help were more likely to be stressed than those who had not; however, this is likely to be a cause-and-effect relationship. It also found that third-year students were more likely to be stressed about reduced holidays, difference in opinions between staff concerning procedures and communicating and approaching staff. Students in higher years were also more often stressed about the transition from pre-clinical to clinical, as were students who worked. First-year students more often considered learning manual dexterity a source of stress, as did single students. Single students were also more likely to be stressed about the difficulty of course work, as were first years and students under the age of 21. Females were more likely to be stressed about moving away from home.

Stress is a main factor in many depressive disorders and can have a detrimental effect on a persons’ life. Undergraduate BDS students have high levels of contact hours as well as unique stress’ in regard to performing procedures on patients at undergraduate levels. This information could be used by dental educators to ensure that students in risk groups are encouraged to seek help if they need it. However, further research is indicated, which could help gain a better understanding of dental students’ stress and consequently improve the stress levels of students to enrich the BDS learning environment.

## Acknowledgements

The authors would like to show appreciation to those staff and students who helped or participated in this project.

## Authors’ contributions

SA and BH designed the study. SA, NR, LAS, DK and YHG collected data. DK, YHG and BH analyzed data. SA, NR, LAS, DK, YHG and BH drafted and edited the manuscript. All the authors have read and approved the final manuscript.

## Funding

The authors did not receive financial support for this research project from any parties.

## Competing interests

The authors declare no competing interests with regards to the authorship and/or publication of this article.

## Ethics approval

Appropriate ethics approval was obtained from the James Cook University (JCU) Human Research Ethics Committee (Reference ID: H5617).
